# Maternal cardiorespiratory fitness is associated with metabolic hormone concentrations in human milk

**DOI:** 10.1038/s44437-026-00010-5

**Published:** 2026-09-29

**Authors:** Emily Rose Ashby, Maëliss Cynthia Chloé Lemoine, Guro F. Giskeødegård, Trine Moholdt

**Affiliations:** 1 Department of Circulation and Medical Imaging, Norwegian University of Science and Technology, Trondheim, Norway; 2 Department of Public Health and Nursing, Norwegian University of Science and Technology, Trondheim, Norway; 3 Department of Gynecology and Obstetrics, St. Olav’s Hospital, Trondheim, Norway

**Keywords:** Biochemistry, Biomarkers, Endocrinology, Health care, Medical research, Physiology

## Abstract

The composition of human milk reflects maternal cardiometabolic health, of which cardiorespiratory fitness is a key determinant. Yet, the influence of maternal fitness on milk-borne hormones remains largely unexplored. In this cross-sectional study, we measured peak oxygen uptake during a maximal exercise test in 149 lactating participants and quantified human milk adiponectin and insulin concentrations using enzyme-linked immunosorbent assays. Higher maternal cardiorespiratory fitness was associated with lower concentrations of milk adiponectin after adjustment for body composition and weeks since delivery (*β* −0.02, 95% CI −0.04 to −0.001). Milk insulin concentrations were inversely associated with fitness after adjustment for body mass index (*β* −0.02, 95% CI −0.04 to −0.001). This work identifies maternal cardiorespiratory fitness as a previously unrecognised factor associated with human milk hormone concentrations, providing a potential pathway linking maternal physiology with early-life metabolic development.

## Introduction

Human milk is recognised as the optimal source of nutrition for infants, providing essential nutrients and bioactive molecules that support growth, metabolic development and immune protection^1,2^. Among these bioactive components are numerous metabolic hormones such as adiponectin, insulin, insulin-like growth factor-1, leptin, and ghrelin^3^. Metabolic hormones present in human milk are primarily derived from maternal circulation^4,5^, though evidence suggests that some may also be synthesised locally within mammary epithelial cells^6^. Of these hormones, adiponectin and insulin have received particular attention due to their well-established roles in energy balance, appetite regulation, and substrate metabolism in adults^7,8^, and accumulating evidence indicates that they may also play an important role in regulating infant metabolism and body composition^9^. Observational studies have reported inverse associations between milk adiponectin and insulin concentrations and infant growth trajectories^10^. However, findings remain inconsistent, and the determinants of these hormone concentrations in human milk are not fully understood^10^.

One potential explanation is that the concentrations of adiponectin and insulin in human milk partly reflect maternal circulating levels^11^, suggesting maternal cardiometabolic health may play an important role in shaping milk composition. Cardiometabolic health encompasses the overall health status of the metabolic and cardiovascular systems. Previous research has shown that modifiable lifestyle factors associated with cardiometabolic health, including diet and body composition, can influence hormone concentrations in human milk^11,12^. Exercise is a well-established lifestyle behaviour known to improve cardiometabolic health^13^. Recently, our group demonstrated that acute exercise can alter milk hormone concentrations: high-intensity interval training increased human milk adiponectin 1 h post-exercise^14^, while endurance exercise had no significant effect on milk insulin concentrations but attenuated postprandial milk insulin responses^15^.

Cardiorespiratory fitness (CRF), measured as peak oxygen uptake (VO_2_peak), is an important physiological indicator of cardiometabolic health and reflects the integrative capacity of the circulatory, respiratory, and muscular systems to deliver and utilise oxygen during sustained exercise^16^. CRF is a strong, modifiable indicator of overall health and a better predictor of morbidity and mortality than physical activity levels alone^17^. Given that CRF influences metabolic processes^18^, it may also modulate circulating hormone concentrations associated with cardiometabolic health^19,20^. For instance, circulating insulin concentrations are inversely associated with CRF^21,22^, whereas adiponectin has shown both positive^23,24^ and inverse^25,26^ associations. Nevertheless, the association between maternal CRF and human milk hormone composition has not yet been investigated.

Given the central role of CRF in metabolic regulation, we aimed to determine whether maternal CRF is associated with human milk concentrations of adiponectin and insulin. Because these hormones may partly reflect maternal circulating concentrations^11^, we hypothesised that higher maternal CRF would be associated with lower milk insulin concentrations and altered adiponectin concentrations.

## Results

We included 149 participants who completed CRF testing and provided a human milk sample. Table 1 shows baseline characteristics for the pooled cohort. The majority of participants were of Norwegian ethnicity (88.7%, *n* = 134). Among participants enrolled in the cross-over studies, fitness testing and body composition measurements were conducted at an average of 7.6 weeks postpartum (standard deviation (SD) 1.8), with human milk collected an average of 2.4 weeks later (SD 0.9).

**Table 1:** Characteristics of participants

	All participants (*N* = 149)	
	Mean (SD)	Range (min–max)
Age (years)	32.7 (3.9)	24.6–43.9
Postpartum (weeks)	10.8 (1.1)	6.3–13.3
Infant birth weight (g)	3644 (424.6)	2825–5800
Infant sex, *n* (female/ male)	77/74	-
Body composition		
Body mass (kg)	74.7 (12.7)	51.9–131.2
Body mass index (kg/m^2^)	26.3 (4.8)	18.5–49.4
Muscle mass (kg)	28.0 (3.0)	20.2–36.9
Visceral fat area (cm^2^)	112.2 (53.9)	22.2–262.8
Fat mass (kg)	24.1 (10.5)	6.1–69.1
Fat percent (%)	31.2 (8.6)	9.5–52.7
Cardiorespiratory fitness testing		
Peak oxygen uptake (mL kg^-1^ min^−1^)	37.9 (7.1)	21.6–56.6
Peak oxygen uptake (L·min^−1^)	2.8 (0.4)	1.73–4.01
Heart rate maximum (beats/min)	188.8 (8.1)	169–214
Maximum respiratory exchange ratio^a^	1.2 (0.04)	1.05–1.26
Rate of perceived exertion^a^	18.7 (2.3)	16–20
Human milk hormones		
Adiponectin (ng/mL)	6.0 (4.3, 7.7)	1.5–18.3
Insulin (μIU/mL)	9.3 (6.7, 15.3)	2.6–125.5

Baseline characteristics and cardiopulmonary exercise testing data are presented as means and standard deviations (SDs) or frequencies (*n*), and ranges (minimum and maximum). Human milk hormone concentrations are reported as medians and 25- and 75-percentiles (quartiles). Rate of perceived exertion according to the Borg 6-20 scale^40^.

^a^Missing data for one participant. Postpartum (weeks) calculated from timepoint since delivery and timepoint for milk collection.

### Associations between VO_2_peak and human milk adiponectin

**Fig. 1:**
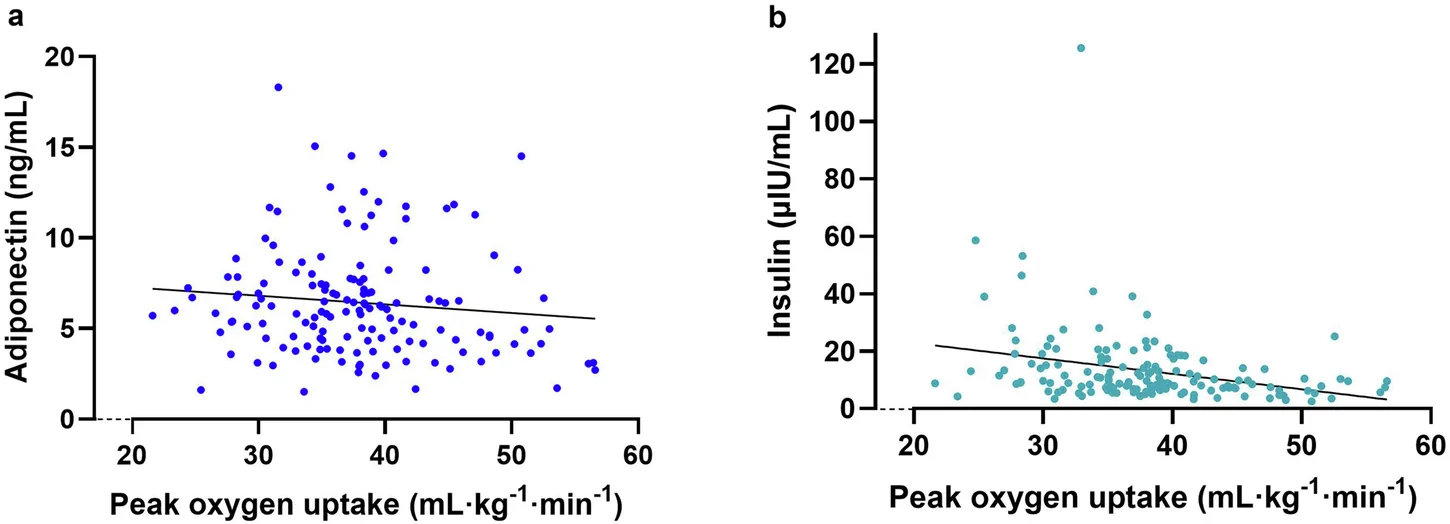
Associations between peak oxygen uptake and human milk metabolic hormones. Panels show log-transformed human milk adiponectin (**a**) and insulin (**b**). Dots represent individual observations. Figures were created using GraphPad Prism 10 (Dotmatics).

Figure 1a shows the individual maternal peak oxygen uptake (VO_2_peak) and adiponectin concentrations for all participants. In unadjusted analysis, VO_2_peak was not statistically significantly associated with human milk adiponectin concentrations (Table 2, model 1). In a multivariate linear regression model adjusting for maternal body mass index (BMI) (Table 2, model 2), VO_2_peak was inversely associated with human milk adiponectin (*β* −0.02, 95% CI −0.03 to −0.004), corresponding to a 1.7% decrease in adiponectin per unit increase in VO_2_peak (in mL·kg^−1^·min^−1^). The inverse association remained significant (*β* −0.02, 95% CI −0.04 to −0.001) when additionally adjusted for fat mass, visceral fat area, and weeks since delivery.

**Table 2:** Regression analysis of associations between maternal peak oxygen uptake (VO_2_peak) and log-transformed human milk adiponectin and insulin concentrations

	Adiponectin		Insulin	
	*Beta* (95% CI)	*P*-value	*Beta* (95% CI)	*P*-value
*Model 1* Peak oxygen uptake, mL·kg^−1^·min^−1^	−0.01 (−0.02, 0)	0.058	−0.03 (−0.05, −0.02)	<**0.001**
*Model 2* Peak oxygen uptake, mL·kg^−1^·min^−1^	−0.02 (−0.03, −0.004)	**0.012**	−0.02 (−0.04, −0.001)	**0.039**
*Model 3* Peak oxygen uptake, mL·kg^−1^·min^−1^	−0.02 (−0.04, −0.001)	**0.036**	−0.01 (−0.03, 0.01)	0.33

*P*-values are from univariate and multivariate linear regression analysis. Model 2 was adjusted for body mass index, and model 3 was adjusted for body mass index, fat mass, visceral fat area, and weeks since delivery. Statistically significant associations (*P* < 0.05) are shown in bold.

Values are beta coefficients (Beta) with 95% confidence intervals (CIs).

### Associations between VO_2_peak and human milk insulin

Figure 1b illustrates individual maternal VO_2_peak and insulin concentrations for all participants. VO_2_peak was inversely associated with human milk insulin concentrations in the unadjusted model (*β* −0.03, 95% CI −0.05 to −0.02), corresponding to a 3.3% decrease per unit increase in VO_2_ peak (Table 2, model 1). In a multivariate linear regression model adjusting for maternal BMI (Table 2, model 2), VO_2_ peak was inversely associated with human milk insulin (*β* −0.02, 95% CI −0.04 to −0.001), corresponding to a 1.8% decrease in insulin per unit increase in VO_2_peak. However, the association was not statistically significant when further adjusted for fat mass, visceral fat, or weeks since delivery (Table 2, model 3). Expected multicollinearity was observed among adiposity-related covariates (BMI, fat mass, and visceral fat area), while variance inflation factors (VIF) for VO_2_peak remained within acceptable limits.

### Exploratory analysis between low vs high CRF and healthy vs high BMI

**Fig. 2:**
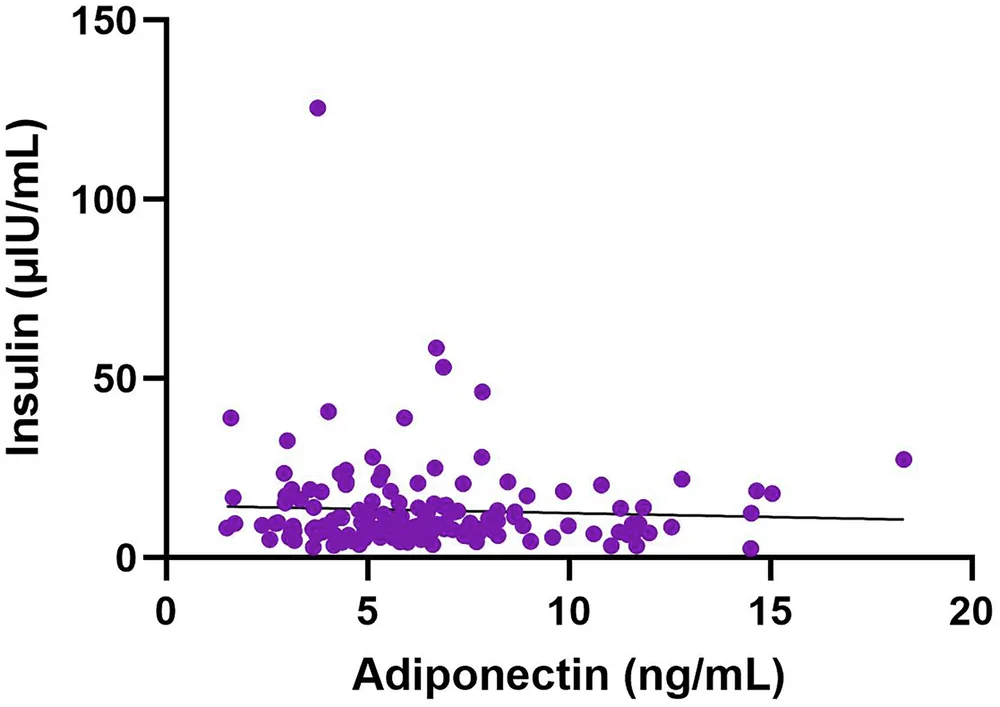
Correlation between human milk adiponectin and insulin. Figure created using GraphPad Prism 10 (Dotmatics).

As shown in Table 3, human milk insulin concentrations were significantly higher in participants with high versus healthy BMI and in those with low versus high VO_2_peak. In contrast, milk adiponectin concentrations did not differ across BMI or VO_2_peak categories. Human milk adiponectin and insulin concentrations were not correlated (*r* = −0.01, *P* = 0.90) (Fig. 2) and the removal of one extreme insulin value did not make it so (*r* = 0.00, *P* = 0.95).

**Table 3:** Human milk adiponectin and insulin concentrations according to maternal body mass index (healthy vs. high BMI) and peak oxygen uptake (high vs. low VO_2_peak)

BMI				
	Healthy (*n* = 68)	High (*n* = 83)	Effect estimate (95% CIs)	*P*-value
Adiponectin	5.9 (4.3, 7.7)	6.1 (4.4, 7.5)	−0.14 (−0.46, 0.19)	0.41
Insulin	8.0 (6.1, 10.3)	12.2 (7.9, 20.3)	−0.76 (−1.09, −0.42)	**<0.001**

VO_2_peak				
	High (*n* = 44)	Low (*n* = 107)	Effect estimate (95% CIs)	*P*-value
Adiponectin	4.9 (3.7, 6.6)	6.3 (4.5, 7.7)	0.32 (−0.04, 0.67)	0.08
Insulin	7.7 (5.2, 9.9)	10.9 (7.3, 18.4)	0.75 (0.39, 1.11)	**<0.001**

Numbers are medians and 25- and 75-percentiles (quartiles) with effect estimates (Cohen’s *d*) and corresponding 95% confidence intervals (CIs) and *P*-values. Effect estimates, 95% CIs, and *P*-values and are from independent samples *t-*tests based on comparisons using log-transformed values. Statistically significant associations (*P* < 0.05) are shown in bold. Equal variances not assumed for body mass index comparisons for log insulin.

*BMI* body mass index, *VO*_2_*peak* peak oxygen uptake.

In sensitivity analyses, the interaction between VO_2_peak and study design was not significant for milk adiponectin (*β* −0.004, 95% CI −0.03 to 0.02) and insulin (*β* −0.01, 95% CI −0.04 to 0.02). There was also no significant main effect of study design (adiponectin= *β* −0.05, 95% CI −0.93 to 0.83, insulin= *β* 0.48, 95% CI −0.67 to 1.61), indicating that the elongated period between milk sampling and the CRF test in the cross-over trials did not affect the results.

## Discussion

This cross-sectional study examined associations between maternal CRF and human milk concentrations of adiponectin and insulin. To our knowledge, this is the first investigation to link maternal CRF with these two key metabolic hormones in human milk. We found a significant inverse association between CRF and adiponectin concentrations after adjusting for BMI, fat mass, visceral fat area, and weeks since delivery. CRF was also inversely associated with human milk insulin concentrations after adjusting for BMI alone, although this relationship was attenuated when additional covariates were included.

CRF is a robust marker of cardiometabolic health, influenced by physical activity, genetics, age, body composition, sex, and smoking status^27^. Reference data from the Trøndelag Health Study (HUNT 3) indicate mean VO_2_peak values of 43.0, 40.0, and 38.4 mL·kg^−1^·min^−1^ in women aged 20–29, 30–39, 40–49, respectively^28^. In our cohort, approximately 70% of postpartum participants exhibited lower CRF than age- and region-matched counterparts, consistent with evidence of reduced fitness up to 1 year postpartum^29^, likely due to physiological and behavioural changes during and after pregnancy^30^. Given that low CRF is associated with adverse cardiometabolic profiles^31^ and is a stronger predictor of morbidity and mortality than anthropometric measures alone^32^, understanding its relevance for lactation-related physiology has important clinical implications.

The observed inverse relationship between CRF and human milk adiponectin suggests that maternal fitness may influence milk adiponectin through mechanisms independent of maternal adiposity, potentially reflecting systemic metabolic adaptations associated with improved fitness. Prior studies in men and adolescents have reported negative associations between circulating adiponectin and fitness^25,26^, consistent with our findings. Although we did not measure maternal circulating adiponectin, the alignment with systemic patterns raises the possibility that higher maternal CRF is accompanied by metabolic adaptations that extend to the mammary gland. These findings highlight maternal fitness as a potential determinant of the hormonal milieu of human milk.

Human milk insulin showed a similar inverse association with CRF adjusted for BMI, consistent with the established link between higher fitness, improved insulin sensitivity, and lower circulating insulin concentrations^21,33^. Elevated milk insulin concentrations have been reported in mothers with type 2 diabetes mellitus compared with BMI-matched mothers with gestational diabetes or normal glucose tolerance^34^, likely reflecting maternal hyperinsulinemia^4^. The attenuation of the CRF-milk insulin association after adjustment for fat mass, visceral fat area, and weeks since delivery indicates that maternal adiposity and weeks postpartum may play stronger roles in shaping milk insulin concentrations. Indeed, visceral adiposity is a stronger predictor of insulin resistance in persons without diabetes than CRF^35^, but its relevance for human milk hormone composition is yet to be elucidated. Further studies incorporating detailed body composition phenotyping, including distribution and metabolic activity of fat deposits, are needed to clarify pathways influencing lactational insulin secretion or mammary transfer.

Exploratory analyses further showed higher milk insulin concentrations in participants with low vs. high CRF and in those with high vs. healthy BMI, reinforcing the notion that maternal metabolic health influences milk insulin levels. We observed no association between milk adiponectin and insulin concentrations. In circulation, adiponectin enhances insulin sensitivity, and the two hormones are typically inversely related in healthy adults^36^. The absence of this relationship in milk may reflect different concentration gradients between blood and milk, as milk insulin concentrations are generally higher than in maternal plasma, whereas adiponectin concentrations are lower^37^. These differences may reflect distinct mechanisms governing the transfer and secretion of hormones into human milk, including contributions from maternal circulation and local production within the mammary gland, suggesting that milk hormone concentrations are not solely determined by maternal circulating levels^6^.

Human milk adiponectin and insulin have been implicated in infant growth and metabolic development, although findings remain inconsistent. Previous studies have reported associations between these hormones and infant growth trajectories, suggesting that variation in milk hormone concentrations may contribute to shaping the early postnatal metabolic environment^9,10^. However, infant outcomes were not assessed in the present study, and therefore the clinical significance of the observed differences in milk hormone concentrations remains unclear. Emerging evidence also suggests that maternal exercise may influence offspring metabolic health through exercise-induced changes in human milk composition beyond adiponectin and insulin concentrations, including alterations in other bioactive milk components^38^. Nevertheless, the mechanisms underlying these effects remain incompletely understood, and whether the associations observed in the present study have implications for infant development requires further investigation.

Our study has several limitations that should be considered. We did not measure circulating maternal hormone levels, constraining our ability to directly link milk hormones to systemic physiology. Another limitation is that we only quantified adiponectin and insulin concentrations, and future studies should include a broader panel of metabolically relevant milk hormones, such as leptin. Additionally, only a single milk sample was collected from each participant, preventing assessment of within-individual variability in hormone concentrations across lactation. Differences in milk sampling protocols across studies within the pooled dataset may also have introduced variability in hormone measurements. Furthermore, the interval between fitness testing and milk collection differed across cohorts, although sensitivity analyses and adjustment for weeks since delivery did not materially affect the primary associations and supported the validity of pooling data across studies. Physical activity data were collected using different assessment tools across cohorts and could therefore not be harmonised for pooled analyses. In addition, medication use data were only available for two of the included studies. Consequently, potential residual confounding by habitual physical activity and medication use cannot be excluded. BMI was used as a proxy measure of adiposity in our analyses; however, it does not directly assess body composition. Analyses using alternative measures yielded similar results, likely reflecting the strong correlation between BMI and more direct adiposity indices in this cohort. Fitness levels before pregnancy and during gestation may influence milk hormone composition, and because these measurements were not available in our participants, we could not account for these factors. Mode of delivery and milk production volume may also influence human milk composition; however, these data were not collected consistently across cohorts and could therefore not be examined in the present study. The predominantly Norwegian, highly educated, and relatively healthy composition of the sample, as well as the inclusion of only participants who were exclusively breastfeeding, may limit the generalisability of these findings to more diverse populations and to individuals with poorer cardiometabolic health. As the first study to examine associations between maternal CRF and human milk adiponectin and insulin concentrations, these findings should be considered hypothesis-generating and require confirmation in independent and longitudinal cohorts.

In summary, we report that higher maternal CRF was associated with lower human milk adiponectin, independent of maternal adiposity and weeks since delivery. CRF was inversely associated with human milk insulin when adjusting for BMI alone, but this association was not independent of additional covariates of fat mass, visceral fat area, and weeks since delivery. These findings advance our understanding of how maternal cardiometabolic health shapes human milk composition and suggest that improving CRF may influence the hormonal environment experienced by the breastfed infant. Longitudinal and interventional studies are now needed to determine whether enhancing maternal fitness postpartum can beneficially modulate milk hormone profiles and support both maternal and infant metabolic health.

## Methods

### Study design and setting

This cross-sectional study pooled data from three independent studies carried out at the Norwegian University of Science and Technology (NTNU) in Trondheim, Norway. The Regional Committee for Medical and Health Research Ethics, Central Norway (REK), approved all studies (REK-263493, 551616, 562012), and this study was conducted in accordance with the Declaration of Helsinki. All participants provided written informed consent prior to participation. We derived data from one cross-sectional study (*n* = 102), and baseline data from two cross-over trials (*n* = 19 and *n* = 28). A small subset of data from one of the cross-over trials (*n* = 19) has previously been published elsewhere^14,15^. However, the analyses presented in the current manuscript are novel. All remaining data have not been published previously. In the cross-sectional study, milk samples and maternal health assessments (including body composition and CRF testing) were obtained on the same day. In the cross-over trials, milk samples and maternal health assessments were conducted on separate days. To ensure comparability between studies, we analysed milk samples collected closest to 10–12 weeks postpartum across all cohorts.

Participants were recruited between February 2021 and August 2025 through targeted social media advertisements and word of mouth. Eligibility screening was conducted by telephone or email. Inclusion criteria for the cross-sectional pooled data study were: (1) ability to complete an exercise test to exhaustion, (2) exclusive breastfeeding of a singleton term infant, (3) 18 years or older, and (3) 9–12 weeks postpartum. One of the cross-over trials included participants 6–12 weeks postpartum; the other included participants 5–12 weeks postpartum and required a BMI of >25 kg/m^2^. Exclusion criteria for all three studies were known cardiovascular disease or type 1 or 2 diabetes. Reporting followed the Strengthening the Reporting of OBservational studies in Epidemiology (STROBE) guidelines^39^.

### Human milk sampling and analysis

The participants were instructed to refrain from high-intensity exercise for 48 h prior to milk collection. On the morning of sampling, participants collected a fasted milk sample before 9:00 using an electric breast pump (Medela Symphony, Switzerland) or a personal pump following proper sterilisation according to manufacturer guidelines. We instructed the participants to express milk from one breast until empty. If <25 mL was obtained, milk could also be expressed from the opposite breast. Samples were gently mixed to minimise variability due to foremilk-hindmilk differences. A 25 mL aliquot was transferred into a sterile Falcon tube and transported on ice packs to the laboratory, where samples were immediately aliquoted and stored at −80 °C until analysis. One of the cross-over studies employed a slightly modified collection protocol, as previously described^15^. In short, participants in that study were instructed to provide at least 25 mL of milk, without explicit full expression, and to store the sample in their home freezer until delivery to the laboratory.

Before adiponectin quantification, we thawed the samples and centrifuged at 10,000 × *g* for 60 min at 4 °C to remove the lipid layer. We used the skimmed milk fraction for adiponectin assays, while whole milk was used for insulin measurement, based on pilot testing indicating optimal performance under these conditions. We used enzyme-link immunosorbent assay (ELISA) to determine the concentrations of adiponectin (IBL International GmBH, Germany, Catalogue no: 30126762) and insulin (IBL International GmBH, Germany, Catalogue no: RE53171). The intra- and inter-assay coefficient of variation were <5% and 7.5% for adiponectin, and <3% and 6% for insulin as detailed by the manufacturer. We utilised a Dynex DS2 automated ELISA system (Montebello Diagnostics AS, Norway), with DS-Matrix software. Based on internal optimisation, final incubation times for both assays were reduced from 15 min to 12 min. All remaining procedures followed the manufacturer instructions. Adiponectin and insulin were detected in 97% and 100% of the samples, respectively. Adiponectin values below the limit of detection (*n* = 4) were imputed using values derived from the ELISA curve-fit equation.

### Anthropometry, questionnaires and cardiorespiratory fitness testing

We measured the participants’ height using a wall-mounted stadiometer and estimated body composition using multi-frequency bioelectrical impedance analysis (InBody 770, Biospace, Seoul, South Korea). The participants completed questionnaires to report demographic and lifestyle variables, including maternal age, parity, physical activity level (average weekly hours and minutes of vigorous and moderate activity), smoking (yes/no), medication use (during and/or after pregnancy, type), and infant characteristics (birth date, birth weight, and sex).

CRF was assessed with a graded treadmill test (Woodway, Germany) with continuous heart-rate monitoring (Polar, Finland). One participant completed the test on a stationary cycle ergometer (Corival, Lode, Netherlands). Sensitivity analyses excluding this participant did not materially alter the results. VO_2_peak and respiratory exchange ratio were measured via indirect calorimetry (Metalyzer II Portable CPX System, Cortex, Germany) following manufacturer-recommended calibration. After a 10-min warm-up at moderate intensity (rating of perceived exertion 11–13 on a 6–20 Borg scale^40^), we fitted the participants with a face mask (V2 series, Hans Rudolph, USA). Individualised ramp protocols began at the final warm-up intensity and increased every 1–2 min by 0.5–1 km/h or 1–2% for the treadmill test, or 25 W every 30 s on the cycle ergometer, until volitional exhaustion. We report VO_2_peak as some participants did not meet VO_2_max criteria^41^. VO_2_peak was calculated as the mean of the three highest consecutive VO_2_ values, and maximum respiratory exchange ratio was the highest value measured from the three corresponding VO_2_ measurements.

### Statistical methods

Data are presented as means with SDs or medians and interquartile ranges. Associations between maternal VO_2_peak (mL·kg^−1^·min^−1^) and milk concentrations of adiponectin and insulin were examined using univariate and multivariate linear regression. We assessed the residuals and standardised residuals for normality, and log-transformed hormone concentrations to improve distributional assumptions. Multicollinearity among covariates was also assessed using VIF.

Initial linear regression models assessed univariate associations between VO_2_peak (independent variable) and milk hormone concentrations (dependent variable). A subsequent multivariable model was adjusted for maternal BMI, which was selected as the primary adjustment variable because it is a widely used measure of adiposity. A third model additionally adjusted for fat mass, visceral fat area, and weeks since delivery (when the milk was sampled). Regression coefficients were back-transformed (exponentiated [*Y* = *e*^β1^]) as percentage changes for interpretability.

Exploratory analyses compared milk hormone concentrations between participants with a healthy (18.5–24.9 kg/m^2^) and high (≥25.0 kg/m^2^) BMI, and between participants with a low and high CRF (a low CRF was classified as a VO_2_peak lower than the average for age-specific regional reference data^28^) using independent sample *t*-tests (Table 3). These group-based analyses were exploratory and were performed to provide clinically interpretable comparisons alongside the primary regression analyses. Equality of variances was met for three of the four comparisons. Correlation between milk adiponectin and insulin concentrations was evaluated using Spearman’s rho.

As we measured VO_2_peak earlier postpartum in the two cross-over trials compared with the cohort from the cross-sectional study, we evaluated potential effect modification by study group. A linear regression model including VO₂peak, study group (coded as 0= cross-sectional, 1= cross-over), and the interaction term VO₂peak × study group was used to test both main effect of study design and the interaction. Statistical significance was defined as *P* < 0.05. The statistical analyses were conducted using IBM SPSS Statistics version 30 and figures were created using GraphPad Prism 10 (Dotmatics).

## Data Availability

Anonymised datasets were deposited in Zenodo data repository and can be found here: https://doi.org/10.5281/zenodo.20825984.

## Code availability

No custom code beyond standard SPSS analysis scripts was used.
